# Deformation-based morphometry applied to FDG PET data reveals hippocampal atrophy in Alzheimer’s disease

**DOI:** 10.1038/s41598-024-70380-z

**Published:** 2024-08-28

**Authors:** Lars Frings, Ganna Blazhenets, Joachim Brumberg, Alexander Rau, Horst Urbach, Philipp T. Meyer

**Affiliations:** 1 Department of Nuclear Medicine, Medical Center – University of Freiburg and Faculty of Medicine, University of Freiburg, Freiburg, Germany; 2 Department of Neuroradiology, Medical Center – University of Freiburg and Faculty of Medicine, University of Freiburg, Freiburg, Germany

**Keywords:** FDG PET, MRI, Atrophy, Morphometry, Alzheimer’s disease, Diagnostic markers, Neurological disorders

## Abstract

Cerebral atrophy is a key finding in patients with dementia and usually determined on MRI. We tested whether cerebral atrophy can be imaged with FDG PET by applying deformation-based morphometry (DBM). We retrospectively identified 26 patients with a biomarker-supported clinical diagnosis of Alzheimer’s disease (AD) who had received FDG PET on a fully-digital PET/CT system and structural MRI and compared them to 13 healthy elderly controls (HEC). We performed DBM with FDG PET data (FDG-DBM). As a reference standard for determining atrophy we used voxel-based morphometry of MRI data (MRI-VBM). For conventional analysis of hypometabolism, scaled FDG PET scans (reference: brain parenchyma) were compared between groups. Receiver operating characteristic (ROC) analyses were performed. ROI read-outs were tested for associations with cognitive test performance. FDG-DBM showed abnormalities in AD mainly in the bilateral hippocampi. Similarly, MRI-VBM showed hippocampal atrophy. By contrast, conventional FDG PET analysis revealed reduced bilateral temporo-parietal FDG uptake (all *p* < 0.05, FWE-corrected). FDG-DBM measures of the hippocampus significantly separated AD from HEC with an AUC of 0.81; MRI-VBM achieved an AUC of 0.87; the difference between the two ROC curves was not significant (*p* = 0.40). Whereas FDG uptake of the hippocampus did not separate AD from HEC, FDG uptake of the Landau Meta-ROI achieved an AUC of 0.88. Verbal memory was significantly associated with FDG-DBM measures of the hippocampus (*p* = 0.009), but not of the Landau Meta-ROI (*p* > 0.1). The opposite held true for conventional FDG uptake (*p* > 0.1 and *p* = 0.001, respectively). Hippocampal atrophy in AD can be detected by applying DBM to clinical, fully-digital FDG PET. It correlates with cognitive performance and might constitute a biomarker of neurodegeneration that is complementary to conventional FDG PET analysis of regional hypometabolism.

## Introduction

Cerebral atrophy is a key finding in patients with dementia and usually determined on MRI. However, MRI may not (due to contraindications) or not yet be available in individual patients admitted to, e.g., PET imaging. Thus, taking advantage of the improved spatial resolution of fully-digital PET/CT systems, the detection of atrophy (in addition to regional hypometabolism) on FDG PET could aid the diagnostic process.

Observer-independent standard methods for the detection of atrophy in dementia are voxel-based morphometry (VBM)^1^ and deformation-based morphometry (DBM)^2^. Whereas FDG PET is usually not used to assess atrophy, in clinical practice atrophy is often detectable on FDG PET of individual elderly patients. Fully-digital PET/CT systems provide an improved spatial resolution in comparison to older, not fully-digital systems (about 4.0 vs. 6 to 7 mm FWHM, see also^3^) and seem promising for assessing morphologic abnormalities. Patients with Alzheimer’s disease (AD) typically display greater atrophy of the hippocampus (and other structures of the medial temporal lobe) than temporo-parietal cortex^1,4–7^. By contrast, hypometabolism is usually much more pronounced in the temporo-parietal cortex than hippocampus^5–8^.

The idea to perform ‘structural’ analysis with FDG PET data has already been promoted previously by Maldjian and Whitlow^8^. The authors used FDG PET data from multiple sources (several sites and different PET systems of the Alzheimer’s Disease Neuroimaging Initiative, ADNI) and showed that abnormalities of the hippocampus can be detected on FDG PET when the spatial transformation necessary to match a template (reflected by the Jacobian determinant) is compared between AD patients and cognitively unimpaired participants. However, the technique was apparently not highly appreciated by the scientific community and, to the best of our knowledge, no further peer-reviewed journal publications exist on that method. Given the recent development in PET technology, we are convinced that this approach should be revisited. Whereas Maldjian & Whitlow^8^ emphasized usage of modulated normalized FDG PET data when searching for hypometabolism in AD, we here focus on the potential utility of spatial deformation maps alone (a by-product of commonly performed spatial normalization) for detection of atrophy on FDG PET.

We determined atrophy by applying deformation-based morphometry to FDG PET acquired on a fully-digital PET/CT system (FDG-DBM) in patients with AD, thereby utilizing functional imaging data for additional structural analyses. We compared findings to regional atrophy determined by established voxel-based morphometry of structural MRI data (MRI-VBM) and hypometabolism in the same patients. Moreover, we assessed individual atrophy patterns in each patient. We hypothesized brain abnormalities in AD detected by FDG-DBM to be more similar to those detected by MRI-VBM than by conventional FDG PET analyses. We expected to observe greater abnormalities of AD patients, when assessed with FDG-DBM, in the hippocampus than in temporo-parietal cortex, and the opposite, when assessing hypometabolism with conventional FDG PET.

## Methods

### Patients

We retrospectively identified 26 patients from our clinical registry who had (1) received FDG PET on a fully-digital Vereos PET/CT system (Philips Healthcare) between 01/2018 and 09/2021, (2) non-contrast, T1-weighted MRI, (3) amyloid or tau PET indicating AD, and (4) a clinical syndrome conforming with AD. For comparison, 13 healthy elderly controls (HEC) were assessed on the same PET/CT system and MR scanner. The institutional ethics committee approved the study protocol (Application No. 137/19). All participants gave written informed consent to retrospective analyses of their data.

Patients were neuropsychologically tested as part of the clinical work-up with the German version ^9^ of the Neuropsychological Assessment Battery of the Consortium to Establish a Registry for Alzheimer’s Disease (CERAD-NAB)^10^. HEC were tested with the same neuropsychological test battery. Global cognitive performance (Mini-Mental State examination, MMSE), verbal and visual memory recall scores were available from 32, 34, and 33 participants, respectively. For patient and HEC characteristics, see Table 1.

**Table 1:** Characteristics of study participants.

	AD (N = 26)	HEC (N = 13)	Group difference
Age (years); M ± S.D	62.6 ± 7.6	68.3 ± 6.7	*p* < 0.05, t test
Sex (f:m)	12:14	6:7	n.s., chi^2^ test
Years of Education^1^; M ± S.D	13.8 ± 2.9	15.5 ± 2.9	*p* < 0.1, t test
MMSE Score^2^; median and IQR	23 [21, 25]	30 [29, 30]	*p* < 0.05, t test
CERAD-NAB Total Score^3^; M ± S.D	54.4 ± 15.6	89.0 ± 6.1	*p* < 0.05, t test
TIV (mL); M ± S.D	1480 ± 141	1538 ± 136	n.s., t test

MMSE, Mini-mental state examination; CERAD, Consortium to Establish a Registry for Alzheimer’s Disease—Neuropsychological Assessment Battery; TIV, total intracranial volume.

^1^Missing in 3 patients.

^2^Missing in 7 patients.

^3^Missing in 5 patients.

### Data acquisition

All PET images were acquired on a fully digital Vereos PET/CT system (Philips Healthcare) 50 to 60 min after injection of 226.2 ± 47.8 (patients) and 193.8 ± 5.1 (HEC) MBq FDG. Using low- dose CT for attenuation correction, a fully corrected emission dataset was reconstructed with the vendor-specific, line-of-response time-of-flight ordered-subsets 3-dimensional iterative reconstruction algorithm using spherically symmetric basis functions (so-called blob ordered-subset time-of-flight reconstruction; number of iterations, 5; number of subsets, 11; 2-mm gaussian postfiltering; resulting voxel size, 1.0 * 1.0 * 1.0 mm), yielding a reconstructed, isotropic image resolution of approximately 4.5 to 5 mm in full width at half maximum. For details on data acquisition and reconstruction see^11^.

MRI was performed with a 3 T scanner (MAGNETOM Prisma, Siemens Healthcare) with a 64-channel head and neck coil. T1-weighted images were acquired with a 3D magnetization-prepared 180° radio-frequency pulses and rapid gradient-echo (MP-RAGE) sequence (repetition time: 2500 ms, echo time: 2.82 ms, flip angle: 7°, inversion time = 1100 ms, GRAPPA factor = 2, 1.0 mm isotropic voxels, 192 contiguous sagittal slices). In six patients, comparable isotropic MP-RAGE data was available from external institutions.

### Data processing

FDG PET of the HEC group were employed to create a study-specific template in MNI space by normalization to the SPM PET template using the ‘old normalise’ module in SPM12 with default settings: no modulation; source image smoothing, 8; nonlinear frequency cutoff, 25; nonlinear iterations, 16; nonlinear regularisation, 1. Visual inspection after normalization confirmed that the normalization worked well. For both, conventional FDG PET analyses and the novel FDG-DBM, we spatially normalized FDG PET data to this FDG template of the 13 HEC. MRI was hence not used for FDG PET processing. No partial volume correction was applied.

FDG-DBM: We first utilized the ‘old normalise’ module in SPM12 with the following settings: source image smoothing, 8; nonlinear frequency cutoff, 12; nonlinear iterations, 4; nonlinear regularisation, 0.1. The resulting spatial normalization matrix was then imported in the ‘deformations’ module in SPM12 (see the script ‘spm_deformations.m’ of the SPM12 distribution) to create a map of the Jacobian determinants which reflects the spatial deformations needed to match the template. Maps of the Jacobian determinants have been constructed such that higher values may be interpreted as less atrophy, similar to gray matter volume (GMV) maps from MRI-VBM (see below).

MRI-VBM: CAT12 was used with default settings to segment and spatially normalize MR images (https://neuro-jena.github.io/cat). Modulated normalized gray matter volume (GMV) maps were smoothed with a Gaussian filter of 12 mm FWHM.

Conventional FDG: We used the ‘old normalise’ module in SPM12 with default settings: no modulation; source image smoothing, 8; nonlinear frequency cutoff, 25; nonlinear iterations, 16; nonlinear regularisation, 1. Normalized images were smoothed with a Gaussian filter of 12 mm FWHM and scaled to the average FDG uptake in the entire brain parenchyma. Both, MRI and FDG PET for conventional analyses, were smoothed with an identical filter to achieve a similar smoothness as observed in Jacobian determinant maps. Smoothness of the data, as estimated by SPM, was 24–26 mm FWHM for FDG-DBM deformation maps and (after smoothing) 18–19 mm for MRI and 19–22 mm FWHM for FDG PET images.

### Statistical analyses

Univariate linear regressions identified significant effects of sex and total intracranial volume (TIV; obtained from MRI processing with CAT12) on measures of FDG-DBM and MRI-VBM (hippocampus from the SPM Anatomy toolbox/Julich Brain Atlas^12^ and Landau Meta-ROI (http://adni.loni.usc.edu/methods/research-tools; see below), leading to selection of these covariates for analyses of FDG-DBM and MRI-VBM. As age has an established effect on hippocampal volume^13^, it was additionally included as covariate. For conventional FDG measures, only age had a significant effect and was therefore selected as covariate in conventional FDG analyses.

(A) We applied the following analyses in SPM in a voxel-wise fashion on the whole brain. Resulting statistical contrasts were explored at uncorrected *p* < 0.001 and cluster extent (k) > 100 voxels (corresponding to 0.1 mL volume) as well as FWE-corrected *p* < 0.05 and k > 100 voxels in all three analyses.

(A1) For FDG-DBM, we applied ANCOVA in SPM with covariates age, sex, and TIV on Jacobian determinants maps to determine greater deformations in AD contrasted with HEC.

(A2) As a reference standard for determining atrophy with MRI-VBM, GMV maps were compared between groups by ANCOVA with age, sex, and TIV as covariates.

(A3) For a conventional analysis of hypometabolism in AD, preprocessed FDG PET were subjected to ANCOVA with covariate age.

(B) We also statistically compared each patient with the group of HEC to obtain individual patterns of greater deformations (FDG-DBM), reduced GMV (MRI-VBM), and hypometabolism (whole brain, voxel-wise analyses). Resulting individual t maps were thresholded (*p* < 0.01, uncorrected, at the voxel level) and binarized. Subsequently, a combined frequency map at the group level of patients with AD was calculated. Furthermore, vectorized t maps (confined to brain parenchyma, but not thresholded) were assessed for similarity at the single subject level between FDG-DBM on one and (a) MRI-VBM and (b) conventional FDG on the other side using Pearson correlation coefficients.

(C1) We performed ROI analyses with read-outs from the Landau Meta-ROI (composite ROI comprising regions within the bilateral posterior cingulate cortex, left and right angular gyrus, and left and right temporal cortex^14^; http://adni.loni.usc.edu/methods/research-tools) and the anatomically defined hippocampus from the SPM Anatomy toolbox/Julich Brain Atlas^12^, adjusted for age (conventional FDG) or age, sex, and TIV (FDG-DBM and MRI-VBM).

Averaged ROI values were tested for pairwise associations between the modalities, FDG-DBM, MRI-VBM, and conventional FDG PET.

Receiver operating characteristic (ROC) analyses were performed to obtain the area under the ROC curve (AUC), sensitivity, and specificity for differentiating patients from HEC with the respective method. ROC curves of FDG-DBM and MRI-VBM of the hippocampus were statistically compared using DeLong’s test.

(C2) ROI read-outs were also tested for associations with cognitive test performance on the MMSE as well as verbal recall and visual recall using linear regressions. The statistical threshold was set to *p* < 0.05.

Statistical analyses were carried out using SPM12 (v7219) on Matlab R2018b (www.mathworks.com) and R 4.3.0 (www.R-project.org).

### Ethical approval and consent to participate

The present retrospective study was approved by the local ethics committee (Ethics committee, University of Freiburg, Application No. 137/19) and carried out in accordance with the declaration of Helsinki and its later amendments. All participants gave written informed consent to retrospective analyses of their data.

## Results

**Figure 1:**
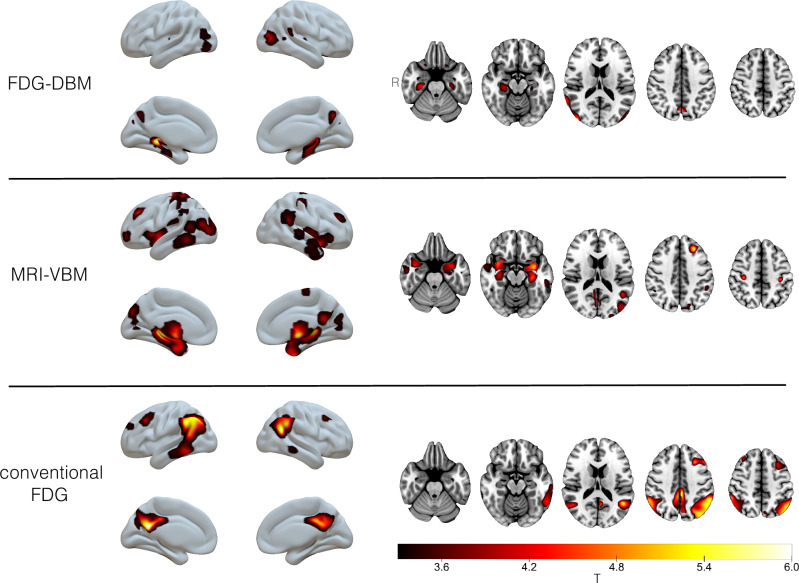
Abnormalities in AD at the group level (compared to HEC; ANCOVA with covariates age, sex, and TIV (FDG-DBM and MRI-VBM) or age (conventional FDG); *p* < 0.001 (uncorrected) and k > 100 voxels (Results surviving FWE-corrected *p* < 0.05 at voxel level are reported in the text).

**Figure 2:**
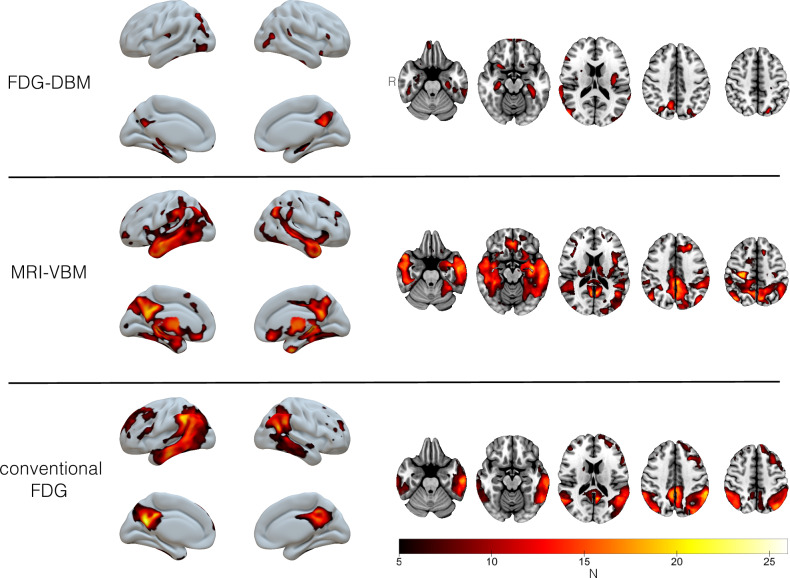
Frequency maps of abnormalities in individual AD subjects (compared with HEC). For display purposes, the contrast showing deficits in individual patients (SPM t map) was thresholded at voxel-level *p* < 0.01 (uncorrected) and binarized such that significant voxels were assigned the value of 1 (all remaining voxels set to 0). Thresholded t maps were combined by summing up; hence, for each modality a deficit map with theoretically possible values from 0 to 26 was obtained. The lower threshold for display was arbitrarily set to voxels with significant abnormality in at least five patients.

Hippocampal abnormalities in AD were observed by both, FDG-DBM and MRI-VBM, but not the conventional FDG analysis. Temporo-parietal abnormalities were only detected by the conventional FDG analysis, but not FDG-DBM:

(A1) Compared to HEC, FDG-DBM of AD patients showed greater local deformation mainly in the medial temporal lobes with maxima in the hippocampi and in the right lateral parieto-occipital cortex (uncorrected *p* < 0.001, k > 100 voxels; Fig. 1 and Supplementary Table S1). A left hippocampal cluster (peak coordinates [−18 −40 −2]) also survived FWE-corrected *p* < 0.05 and k > 100 voxels.

(A2) Similarly, VBM of MRI data showed reduced gray matter volume predominantly in both medial temporal lobes (peak coordinates [26 −32 5]), but also in the left prefrontal cortex (uncorrected *p* < 0.001, k > 100 voxels). Clusters in these three regions also survived thresholding at FWE-corrected *p* < 0.05 and k > 100 voxels.

(A3) In contrast, conventional FDG PET analysis revealed reduced FDG uptake in the bilateral posterior cingulate cortex and lateral temporo-parietal cortices (uncorrected *p* < 0.001 and k > 100 voxels). Clusters in these regions also remained significant after applying a threshold of FWE-corrected *p* < 0.05 and k > 100 voxels.

(B) The single-subject statistical contrasts against HEC mainly recapitulated group level findings (Fig. 2). Importantly, FDG-DBM of the majority of individual patients showed the expected local deformation of the hippocampus and the posterior cingulate cortex: In detail, 20/26 patients had abnormal voxels in the hippocampal ROI (individual statistical comparisons with 13 HEC, thresholded at voxel-level *p* < 0.01). The number of abnormal hippocampal voxels detected by FDG-DBM was negatively correlated with the MMSE score (Pearson’s r = −0.61, *p* < 0.05).

**Figure 3:**
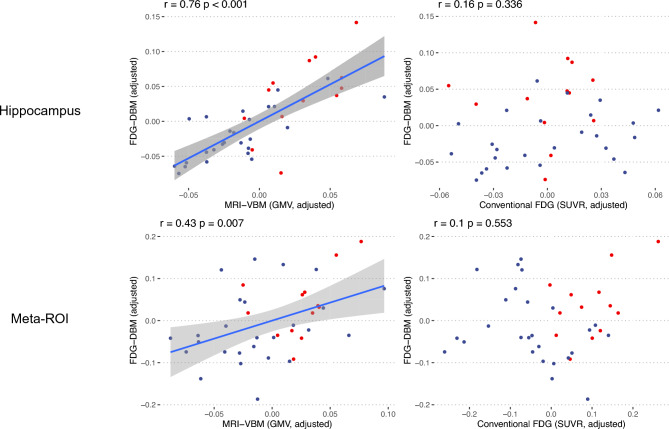
Hippocampal and Meta-ROI values from FDG-DBM against MRI-VBM and conventional FDG (adjusted for age, sex, and TIV in case of FDG-DBM and MRI-VBM, adjusted for age in case of conventional FDG analyses) with linear fit and 95% confidence interval. Blue and red dots represent patient or HEC data, respectively.

The median correlation coefficient between patients’ individual statistical maps from FDG-DBM and MRI-VBM was 0.27 (95% CI 0.23–0.31), whereas it was 0.11 (95% CI 0.05–0.16) for the correlation between FDG-DBM and the conventional FDG analysis. The median intraindividual difference between the two correlation coefficients was 0.16 (95% CI 0.11–0.22).

(C1) ROI analyses showed that hippocampal FDG-DBM values were significantly associated with MRI-VBM (r = 0.76, *p* < 0.001; Fig. 3), but not the conventional FDG values (r = 0.16, *p* = 0.336). Similarly, FDG-DBM values of the Landau Meta-ROI were significantly associated, though to a lesser extent, with MRI-VBM (r = 0.43, *p* < 0.01), but not the conventional FDG values (r = 0.10, *p* = 0.553).

**Figure 4:**
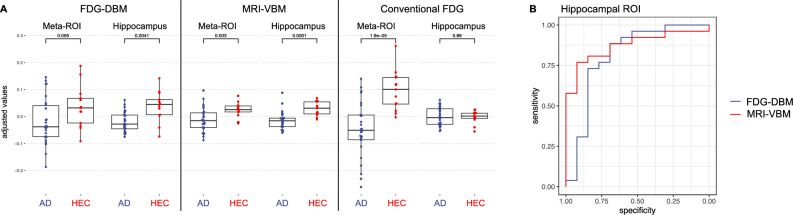
(**A**) values from the Landau Meta-ROI and the hippocampus from the Julich Brain Atlas for each of the three modalities, FDG-DBM, MRI-VBM, and conventional FDG analysis (adjusted). (**B**) ROC curves of hippocampal values from FDG-DBM (AUC = 0.81; *p* = 0.001) and MRI-VBM (AUC = 0.87; *p* < 0.001). The difference between the two was not significant (*p* = 0.40).

FDG-DBM of the hippocampus achieved an AUC, sensitivity, and specificity of 0.81 [0.64–0.98], 0.88, and 0.69, respectively (*p* = 0.001). MRI-VBM of the hippocampus achieved an AUC of 0.87 [0.76–0.98], sensitivity of 0.77 and specificity of 0.92 (*p* = 0.00007). The difference between the latter two ROC curves was not significant (*p* = 0.40; Fig. 4). Whereas FDG uptake of the hippocampus did not separate AD from HEC, FDG uptake of the Landau Meta-ROI achieved an AUC of 0.88 [0.78–0.99], sensitivity of 0.77 and specificity of 0.92 (*p* = 0.00003).

**Figure 5:**
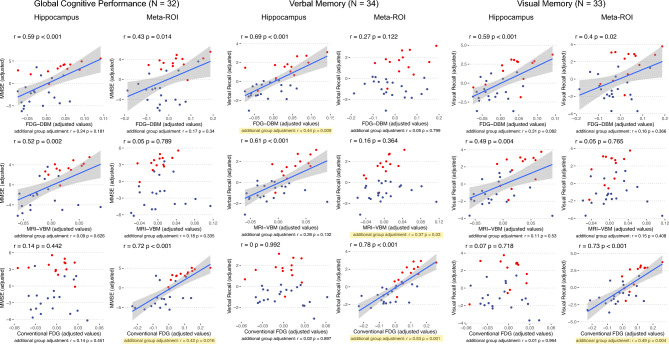
Associations between cognitive test scores and measures of the two ROI for each of the three modalities, FDG-DBM, MRI-VBM, and conventional FDG. Variables were adjusted for age, sex, and TIV (FDG-DBM and MRI-VBM) or age (conventional FDG). *P* and r values below scatter plots were corrected for the additional effect of factor ‘group’ (highlighted in yellow if significant); those above scatter plots were not corrected for the group effect. Blue and red dots represent patient or HEC data, respectively. Blue line shows linear fit, gray area 95% confidence interval.

A post-hoc test of the additive value of FDG-DBM of the hippocampal ROI over FDG uptake of the Landau Meta-ROI showed that both variables combined only slightly improved the ROC-AUC to 0.90 [0.80–1.0] (versus 0.88 for FDG uptake of the Landau Meta-ROI alone, see above). This improvement was not significant (*p* = 0.58, DeLong’s test). Adding MRI-VBM of the hippocampus also did not improve the AUC achieved by FDG uptake of the Landau Meta-ROI alone (0.91 [0.82–1.0], *p* = 0.42).

(C2) The highest associations with MMSE, verbal and visual memory performance of FDG-DBM and MRI-VBM measures were found in the hippocampus (r values of 0.59, 0.69, and 0.59, respectively, for FDG-DBM; r values of 0.52, 0.61, and 0.49, respectively, for MRI-VBM; all survived Bonferroni-correction for 18 tests: *p* < 0.0028), less so in the Meta-ROI (r values of 0.43, 0.27, and 0.4 for FDG-DBM; r values of 0.05, 0.16, and 0.05 for MRI-VBM; all *p* > 0.0028; for details, see Fig. 5). The opposite pattern was observed for conventional FDG uptake, which was highly associated with global cognition, verbal and visual memory regarding the Meta-ROI (r values of 0.72, 0.78, and 0.73, respectively; all *p* < 0.0028), but not the hippocampus (r values of 0.14, 0, and 0.07, respectively; all *p* > 0.1).

After controlling for the group effect (AD vs. HEC), only associations of FDG-DBM measures of the hippocampus (r = 0.44) with verbal memory, MRI-VBM measures of the Meta-ROI with verbal memory (r = 0.37), and conventional FDG uptake of the Meta-ROI with global cognition, visual and verbal memory were significant at exploratory *p* < 0.05 (r values of 0.42, 0.49, and 0.53, respectively; only the latter remained significant when applying Bonferroni-corrected *p* < 0.0028).

A post-hoc test of the additive value of FDG-DBM of the hippocampal ROI over FDG uptake of the Landau Meta-ROI showed that a model including both variables did not improve prediction of global cognition compared to the model including FDG uptake of the Landau Meta-ROI only (R^2^ of 0.765 for both models with covariates age, sex, TIV, and group).

## Discussion

DBM applied to FDG PET data revealed abnormalities in AD patients that are in line with MRI-based VBM as the reference standard method for detection of brain atrophy. Hippocampal values measured with FDG-DBM separated AD cases from HEC with considerable accuracy (AUC = 0.81), which was not statistically inferior to MRI-VBM (AUC = 0.87). Global cognition and memory test performance were correlated with hippocampal measures from FDG-DBM and those from MRI-VBM. Of note, FDG-DBM are easy to implement in an automated fashion and come at no extra charge.

Atrophy is a key finding in patients with dementia. It is regarded as a biomarker of neurodegeneration, e.g. in the NIA-AA research framework for AD^15^. While the reference standard for measuring atrophy is undoubtedly MRI, there are clinical cases who are referred to FDG PET without having yet received MRI or in whom MRI is not possible due to contraindications. In these cases, determination of atrophy with DBM of the same FDG PET might prove valuable for the diagnostic decision. This value is corroborated by a closer look at patients with the seemingly contradicting findings of particularly low MMSE scores and AD-typical hypometabolism but no hippocampal atrophy detected by FDG-DBM (or MRI-VBM): One patient (MMSE score of 21) also had cerebrovascular disease, another (MMSE score of 22) also had REM sleep behavioral disorder. This might suggest that when hypometabolism on FDG PET indicates AD but hippocampal atrophy on FDG-DBM cannot be detected, other conditions/comorbidities beyond AD might contribute to the cognitive impairment, like cerebrovascular disease, or Lewy body disease.

The approach we pursued, detection of atrophy by applying DBM to FDG PET data, is based on the assumption that the morphological information present in FDG PET images can be utilized. Although the spatial resolution is not as fine as that of MRI, we assume FDG PET is capable of imaging morphological abnormalities in disease. Notably, DBM describes only the required deformation for a brain image to be mapped onto a healthy brain. Hence, we did not intend to achieve accurate imaging of brain structures (e.g., the hippocampus), but merely their alteration in disease. One may speculate that FDG-DBM results are not pure measures of atrophy but to a certain degree ‘contaminated’ by hypometabolism of the remaining tissue, just as conventional analyses of FDG uptake convey information about atrophy. However, FDG-DBM results were much more similar to the atrophy detected by MRI-VBM than hypometabolism imaged with conventional FDG-PET (findings by conventional FDG und FDG-DBM virtually were mutually exclusive): Firstly, FDG-DBM-based abnormalities were, at the single-subject level, more closely associated with the reference standard measure of atrophy than with hypometabolism. Moreover, FDG-DBM measures of the hippocampus revealed closer correlations with MRI-VBM than conventional FDG. Secondly, we observed a double dissociation, with conventional FDG analyses revealing the typical parieto-temporal hypometabolism in AD, but none in the hippocampus; by contrast, FDG-DBM showed hippocampal abnormalities in AD, but no temporo-parietal abnormalities, neither in whole-brain analyses nor of the Landau Meta-ROI.

Our observation of hippocampal atrophy in AD when applying DBM to FDG PET data replicates the finding by Maldjian and Whitlow^8^. To the best of our knowledge, the study by Maldjian and Whitlow^8^ is the only one that investigated the utility of FDG PET for measuring atrophy. In contrast to that study, the current study shows hippocampal atrophy with FDG-DBM also at the single-subject level, making it easier to envision its use in clinical routine. To achieve this, we made use of the advantage of a fully-digital PET/CT system, which is capable of imaging at higher spatial resolution than older, not fully-digital systems. Furthermore, we here utilized code from the software package SPM that can be regarded as well-established in the brain imaging community and widely available, which promotes application of the technique in further studies and data sets.

Glucose metabolism of the Meta-ROI (conventional FDG PET) marginally better separated AD from HEC than gray matter volume of the hippocampus (MRI-VBM) (AUC of 0.88 vs 0.87; not statistically significant). This is line with a previous publication^7^ (AUC of 0.99 vs 0.83) which, however, reported better separation by FDG PET compared to our study. FDG-DBM yielded a worse separation (AUC of 0.81), but this separation was still significant, and it was not statistically significantly inferior to the reference standard MRI-VBM.

### Limitations

ROI analyses were restricted to two exemplary regions that play important roles in AD research: the Landau Meta-ROI and the hippocampus. Both were selected a priori, and we assume that even better separation of AD from HEC might be achieved with tailored ROI, e.g., comprising voxels identified in whole brain group contrasts.

Choice of parameters of FDG-DBM with the Deformations module in SPM12 might be optimizable; this was not systematically tested. Atrophy and hypometabolism are both regarded as biomarkers of neurodegeneration, but still, they carry complementary information. Thus, a detailed correlation to AD neuropathological biomarkers (soluble and aggregated amyloid and tau) would be of great interest to unravel their roles. Finally, due to the retrospective nature of the present work, future prospective studies are warranted for additional validation.

## Conclusions

Hippocampal atrophy in AD can be detected by applying DBM to clinical, fully-digital FDG PET. It correlates with cognitive performance and might constitute a biomarker of neurodegeneration that is complementary to conventional FDG PET analysis of regional hypometabolism.

### Supplementary Information

Supplementary Information.

## Data Availability

The data that support the findings of this study are available on request from the corresponding author. The data are not publicly available as they contain information that could compromise the privacy of research participants.

## Abbreviations

AD: Alzheimer’s disease
AUC: Are under the curve
CT: Computed tomography
DBM: Deformation-based morphometry
FDG: Fluorodeoxyglucose
FWE: Family-wise error
HEC: Healthy elderly controls
MMSE: Mini-mental status examination
MRI: Magnetic resonance tomography
PET: Positron emission tomography
ROI: Region of interest
ROC: Receiver-operating characteristic
SPM: Statistical parametric mapping
VBM: Voxel-based morphometry
