# Supplementary material for: Deformation-based morphometry applied to FDG PET data reveals hippocampal atrophy in Alzheimer’s disease
Source: Sci Rep. 2024 Aug 28;14:20030. doi: 10.1038/s41598-024-70380-z (PMC11358471; doi:10.1038/s41598-024-70380-z)
Supplement: Supplementary file 1 — Supplementary Information. [file 41598_2024_70380_MOESM1_ESM.docx]

**SUPPLEMENT**

Supplementary Table 1. Clusters of significant local deformations (FDG-DBM), reduced GMV (MRI-VBM), and hypometabolism (conventional FDG) from group comparisons of 26 AD patients against 13 HEC (ANCOVA; p < 0.001, k > 100 voxels).

| **Cluster level**  **cluster** | | **Peak level** | | | | | **MNI coordinates** | | |
| --- | --- | --- | --- | --- | --- | --- | --- | --- | --- |
| **k (voxels)** | **p (unc)** | **p (FWE-corr)** | **p (FDR-corr)** | **T** | **Z** | **p (unc)** | **x** | **y** | **z** |
| **FDG-DBM** | | | | | | | | | |
| 8138 | 0.02199 | 0.07771 | 0.02628 | 4.7 | 4.1 | 0.00002 | 17 | -35 | 0 |
| 5099 | 0.06111 | 0.04438 | 0.01952 | 5.0 | 4.3 | 0.00001 | 42 | -85 | 13 |
| 4540 | 0.07525 | 0.00251 | 0.01067 | 6.1 | 5.0 | <0.000005 | -18 | -40 | -2 |
| 2671 | p>0.1 | 0.07829 | 0.02635 | 4.7 | 4.1 | 0.00002 | -2 | -70 | 41 |
| 2407 | p>0.1 | p>0.1 | 0.03997 | 3.9 | 3.5 | 0.00020 | -45 | -82 | -12 |
| 442 | p>0.1 | p>0.1 | 0.04515 | 3.7 | 3.4 | 0.00038 | -33 | -20 | 2 |
| 421 | p>0.1 | p>0.1 | 0.04712 | 3.6 | 3.3 | 0.00044 | -19 | 11 | -21 |
| 204 | p>0.1 | p>0.1 | 0.04704 | 3.7 | 3.3 | 0.00043 | -68 | -43 | 4 |
| 166 | p>0.1 | p>0.1 | 0.04785 | 3.6 | 3.3 | 0.00047 | 22 | 15 | -25 |
| 123 | p>0.1 | p>0.1 | 0.04507 | 3.7 | 3.4 | 0.00037 | 41 | -7 | -48 |
| **MRI-VBM** | | | | | | | | | |
| 24444 | 0.00000 | 0.00077 | 0.00044 | 6.8 | 5.4 | <0.000005 | 26 | -32 | 5 |
| 2362 | 0.00352 | 0.23500 | 0.00263 | 4.5 | 3.9 | 0.00004 | -45 | -56 | 14 |
| 1488 | 0.01575 | 0.35759 | 0.00356 | 4.2 | 3.8 | 0.00008 | -38 | -87 | 11 |
| 1082 | 0.03485 | 0.34426 | 0.00346 | 4.3 | 3.8 | 0.00008 | 5 | -53 | 20 |
| 1068 | 0.03588 | 0.07058 | 0.00126 | 5.0 | 4.3 | 0.00001 | -35 | -27 | 50 |
| 900 | 0.05136 | 0.00034 | 0.00044 | 7.1 | 5.5 | <0.000005 | -26 | 30 | 36 |
| 895 | 0.05193 | 0.45962 | 0.00433 | 4.1 | 3.7 | 0.00012 | 62 | -47 | 24 |
| 770 | 0.06886 | 0.26596 | 0.00286 | 4.4 | 3.9 | 0.00005 | -5 | -78 | 33 |
| 488 | p>0.1 | 0.07438 | 0.00130 | 5.0 | 4.3 | 0.00001 | 33 | -24 | 51 |
| 445 | p>0.1 | p>0.1 | 0.00215 | 4.6 | 4.0 | 0.00003 | -32 | 53 | -3 |
| 392 | p>0.1 | p>0.1 | 0.00429 | 4.1 | 3.7 | 0.00012 | 29 | 38 | 30 |
| 286 | p>0.1 | p>0.1 | 0.00682 | 3.7 | 3.4 | 0.00033 | -50 | -18 | 24 |
| 216 | p>0.1 | p>0.1 | 0.00718 | 3.7 | 3.4 | 0.00037 | 59 | -29 | 3 |
| 181 | p>0.1 | p>0.1 | 0.00461 | 4.0 | 3.6 | 0.00014 | 11 | -21 | 69 |
| 152 | p>0.1 | p>0.1 | 0.00704 | 3.7 | 3.4 | 0.00035 | -50 | -42 | 39 |
| **Conventional FDG** | | | | | | | | | |
| 37383 | 0.00000 | 0.01010 | 0.00093 | 5.7 | 4.8 | <0.000005 | -39 | -74 | 44 |
| 18139 | 0.00006 | 0.00259 | 0.00093 | 6.2 | 5.1 | <0.000005 | -5 | -54 | 24 |
| 14917 | 0.00020 | 0.00796 | 0.00093 | 5.8 | 4.8 | <0.000005 | 51 | -59 | 25 |
| 4647 | 0.02008 | 0.08749 | 0.00119 | 4.9 | 4.2 | 0.00001 | -28 | 23 | 40 |
| 811 | p>0.1 | p>0.1 | 0.00810 | 3.8 | 3.4 | 0.00030 | 66 | -40 | -11 |
| 631 | p>0.1 | p>0.1 | 0.00546 | 4.0 | 3.6 | 0.00016 | 34 | 30 | 47 |
| 401 | p>0.1 | p>0.1 | 0.01127 | 3.6 | 3.3 | 0.00050 | -23 | 48 | 30 |
| 192 | p>0.1 | p>0.1 | 0.00695 | 3.8 | 3.5 | 0.00024 | 41 | 18 | 44 |
